# Supplementary material for: Optogenetic control of Neisseria meningitidis Cas9 genome editing using an engineered, light-switchable anti-CRISPR protein
Source: Nucleic Acids Res. 2020 Dec 16;49(5):e29. doi: 10.1093/nar/gkaa1198 (PMC7969004; doi:10.1093/nar/gkaa1198)
Supplement: gkaa1198_Supplemental_Files [file gkaa1198_supplemental_files.zip › CASANOVA_C3_supplements_revision_final.pdf]

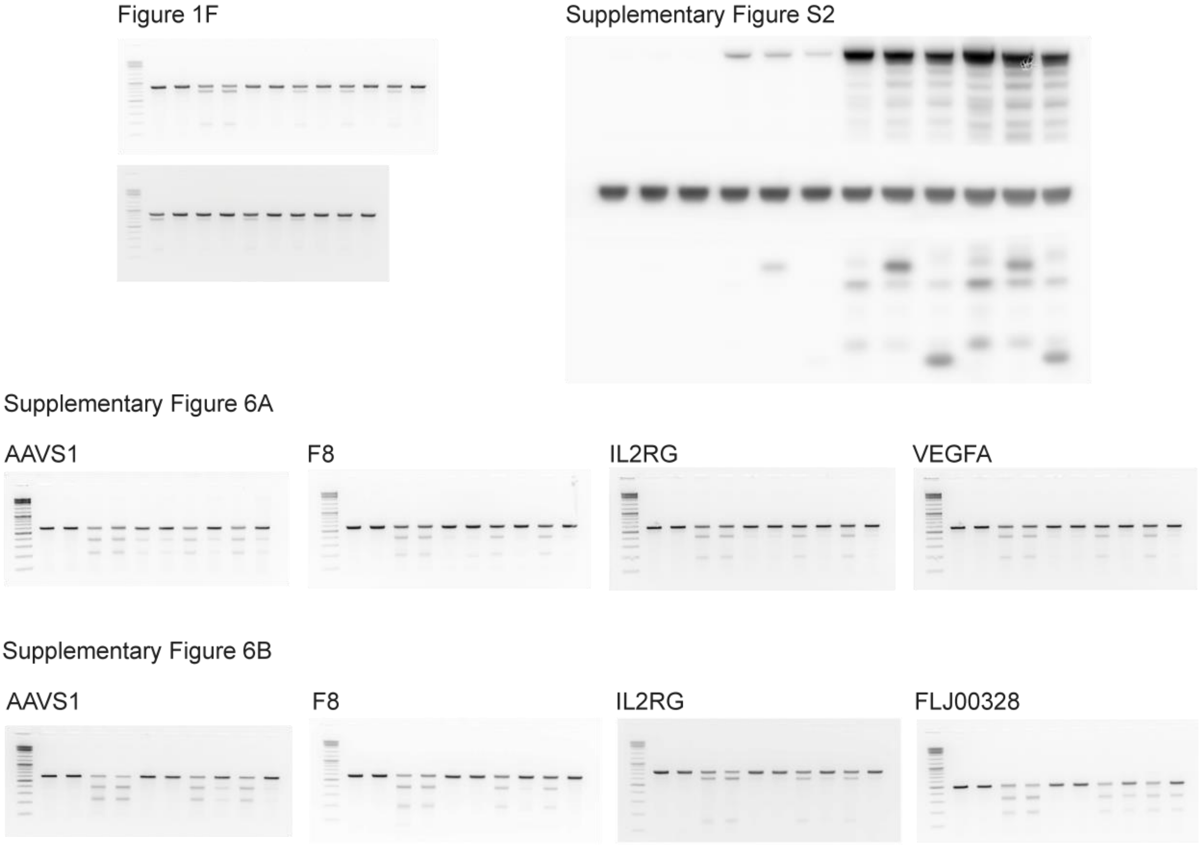

Supplementary Figure S1. Full-length images of T7 agarose gels and Western blots. The Gene Ruler DNA Ladder Mix (Thermo Fisher Scientific) was used in all cases (left lane).

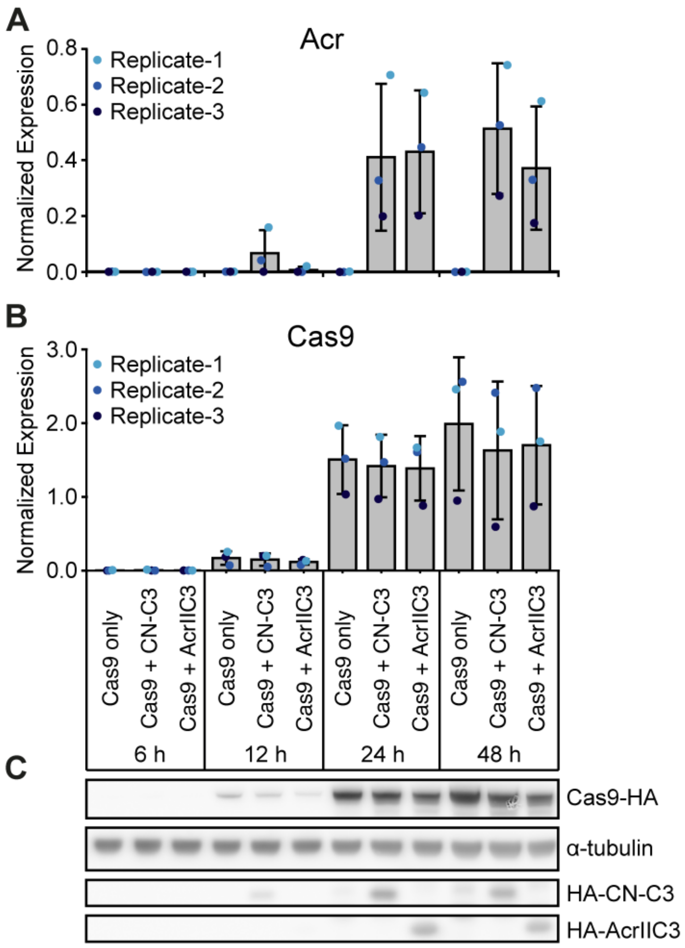

Supplementary Figure S2. Kinetics of *Nme*Cas9 and CN-C3 expression after transient transfection. HEK293T cells were transfected with plasmids encoding *Nme*Cas9, a non-targeting sgRNA and the indicated Acr. A Cas9:Acr plasmid ratio of 1:1 was used. At the indicated time points, the cells were lysed and protein levels were assessed by Western blot. (**A, B**) The relative expression levels of the Acrs (**A**) and *Nme*Cas9 (**B**) were calculated by normalizing the respective band intensities to the α-tubulin reference. Bars represent means, error bars the standard deviation and dots individual data points from n = 3 independent experiments. Individual replicates are color-coded. (**C**) Representative membrane images are shown.

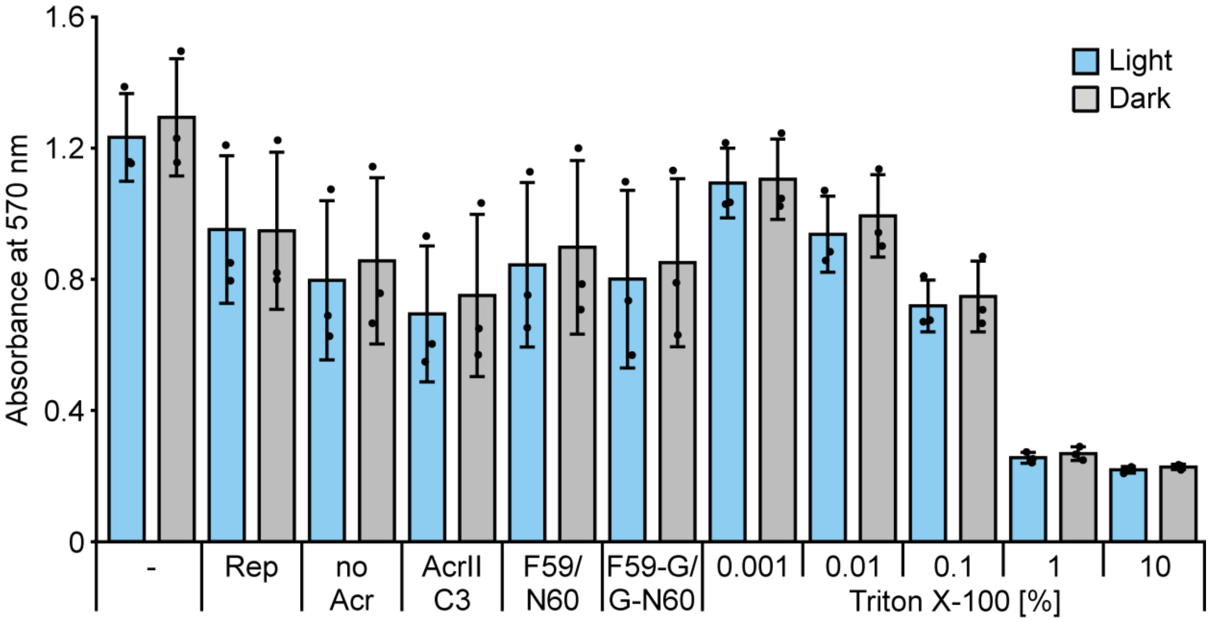

Supplementary Figure S3. MTT assay to assess cell viability in samples irradiated with blue light or incubated in the dark. HEK293T cells were either transfected with constructs encoding (i) a luciferase reporter, (ii) *Nme*Cas9 and a sgRNA targeting the reporter gene and (iii) either wt AcrIIC3 or the indicated AcrIIC3-LOV2 hybrid. Samples were irradiated with blue light or kept in the dark for 48 hours, before the MTT assay was performed. Non-transfected cells served as negative control (dash), while cells treated with different concentrations of Triton (0.001% - 10%) were used as a positive control for cell toxicity. Bars represent means, error bars the standard deviation and dots individual data points from n = 3 independent experiments. Rep, reporter only control.

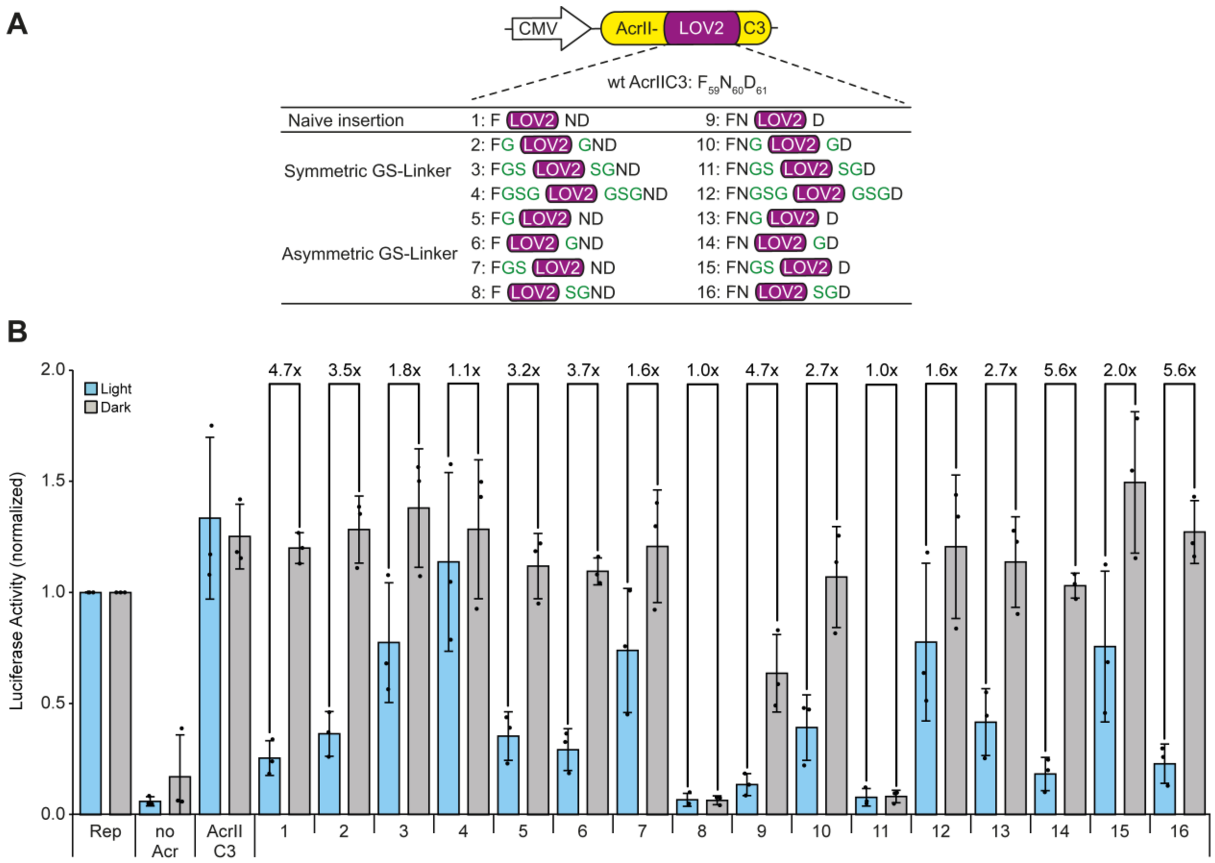

Supplementary Figure S4. Optimization of AcrIIC3-LOV2 hybrids by linker insertion. (**A**) A library of 17 constructs was designed using the variants with the LOV2 domain inserted behind F59 or N60 as scaffold. Short symmetric and asymmetric glycine-serine linkers were included at the Acr-LOV boundaries (indicated in green). (**B**) Luciferase assay-based screening of the variants in **A**. HEK293T cells were transfected with constructs encoding (i) a luciferase reporter, (ii) *Nme*Cas9 and a sgRNA targeting the reporter gene and (iii) the indicated AcrIIC3-LOV2 hybrid. Samples were irradiated with blue light or kept in the dark for 48 hours. Bars represent means, error bars the standard deviation and dots individual data points from n = 3 independent experiments. Rep, reporter only control.

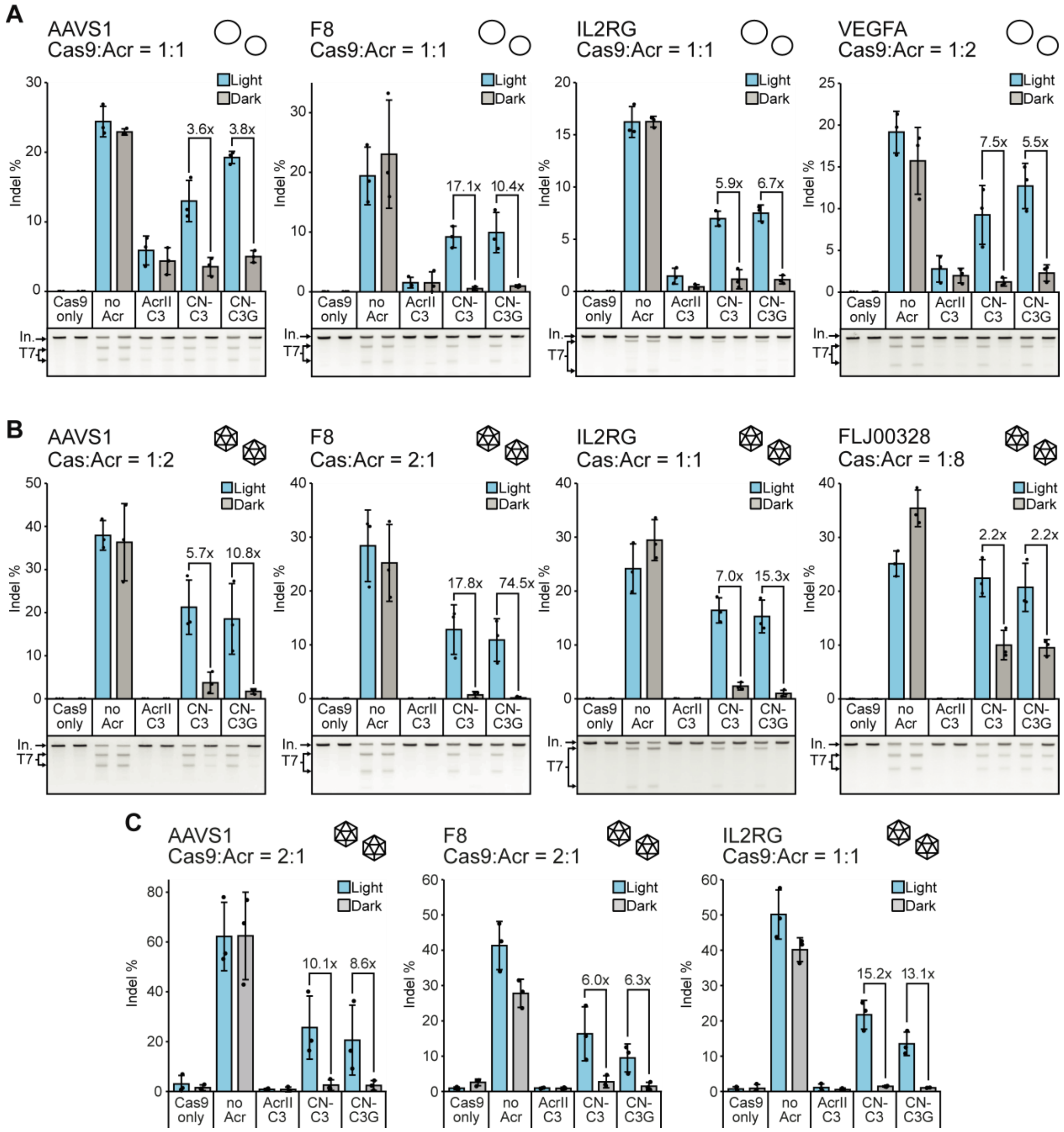

Supplementary Figure S5. CN-C3 and CN-C3G enable light-dependent genome editing of various loci. HEK293T cells were co-transfected with plasmids (**A**) or co-transduced with AAV vectors (**B, C**) encoding (i) *Nme*Cas9 and a sgRNA targeting the indicated locus and (ii) the indicated Acr variant. Cells were then irradiated with pulsed blue light or kept in the dark for 72 hours, followed by T7 assay (**A, B**) or TIDE sequencing (**C**). Cas9:Acr vector mass ratios (**A**) and AAV lysate volume ratios (**B, C**) used during transfection or transduction, respectively, are indicated. Bars represent mean values, error bars the standard deviation and dots individual data points from n = 3 independent experiments. Representative gel images are shown below the bar charts. In, Input; T7, T7 cleavage fragments.

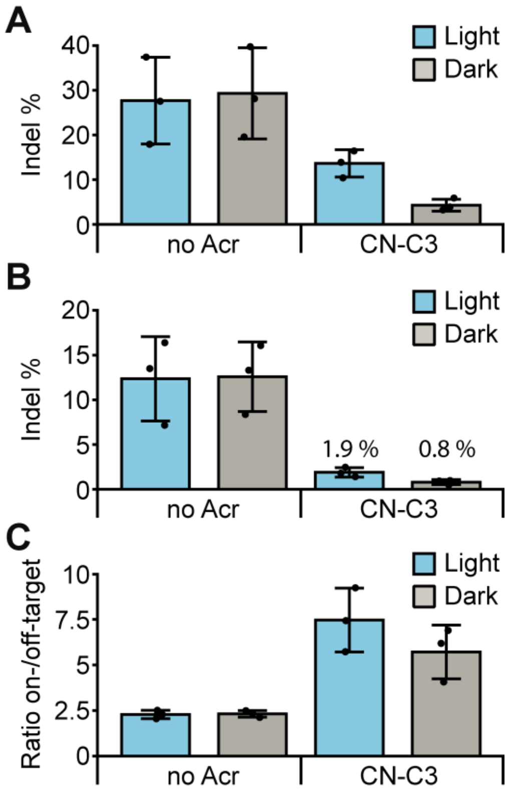

Supplementary Figure S6. CN-C3 reduces off-target editing. (**A, B, C**) HEK293T cells were transfected with (i) a plasmid encoding *Nme*Cas9 and a sgRNA targeting the SLC9A9 (1) locus and (ii) the CN-C3 construct or a stuffer plasmid to keep the transfected amount of DNA constant. 72 hours post-transfection, the cells were lysed and the editing outcomes at the target locus (**A**) and a previously published off-target locus (chr16:+:30756950:30756980 (1)) (**B**) were analysed by targeted amplicon sequencing. (**C**) The specificity, i.e. on-/off-target editing ratio, was higher in presence of CN-C3 as compared to the use of *Nme*Cas9 alone.

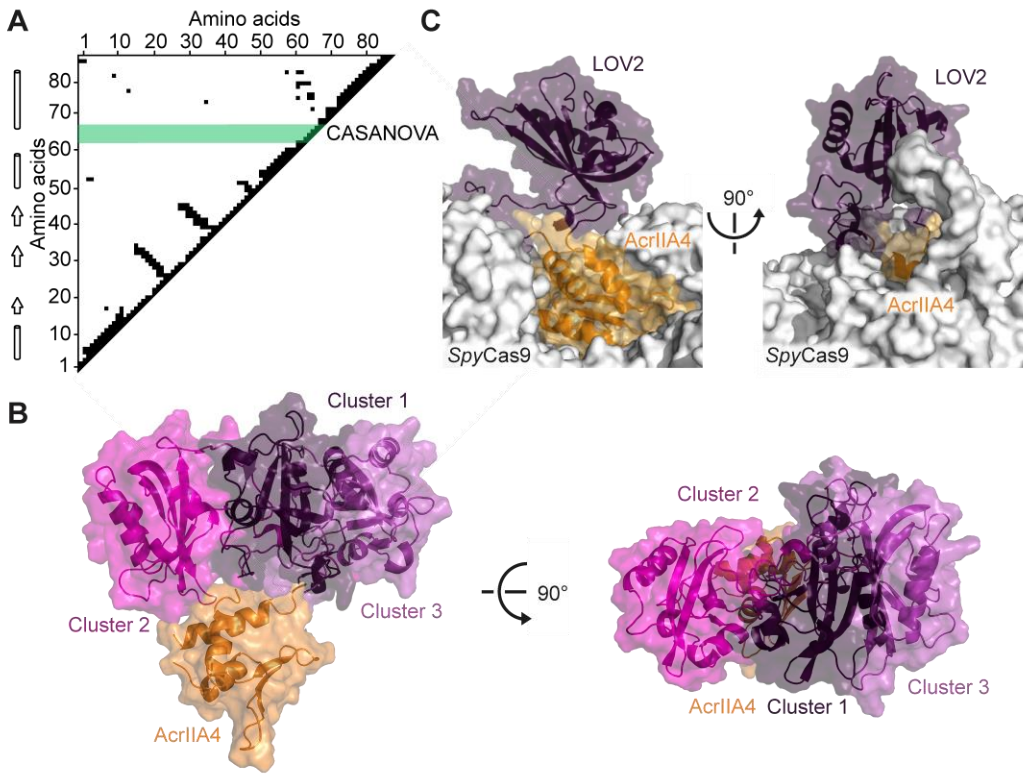

Supplementary Figure S7. Structural analysis of CASANOVA, an optogenetic *Spy*Cas9 inhibitor. The data underlying this figure has been previously reported by us (2) and is shown here to facilitate comparison with CN-C3(G) (Figure 3 and Supplementary Figure S8). (**A**) Analysis of AcrIIA4 residue contacts. Spatially proximate AcrIIA4 residue pairs (distance <7 Å) are indicated by black squares. Secondary structure elements are shown on the left. The region, in which the LOV2 domain was inserted into AcrIIC3 is marked in green. (**B**) Computational model of CASANOVA generated by domain assembly simulation. The three most populated LOV2 conformational clusters are shown in purple in descending order. (**C**) Structural model of CASANOVA bound to *Spy*Cas9. The shown LOV2 configuration corresponds to the cluster 1 in C. (**B, C**) PDB 5VW1 and 2V0W.

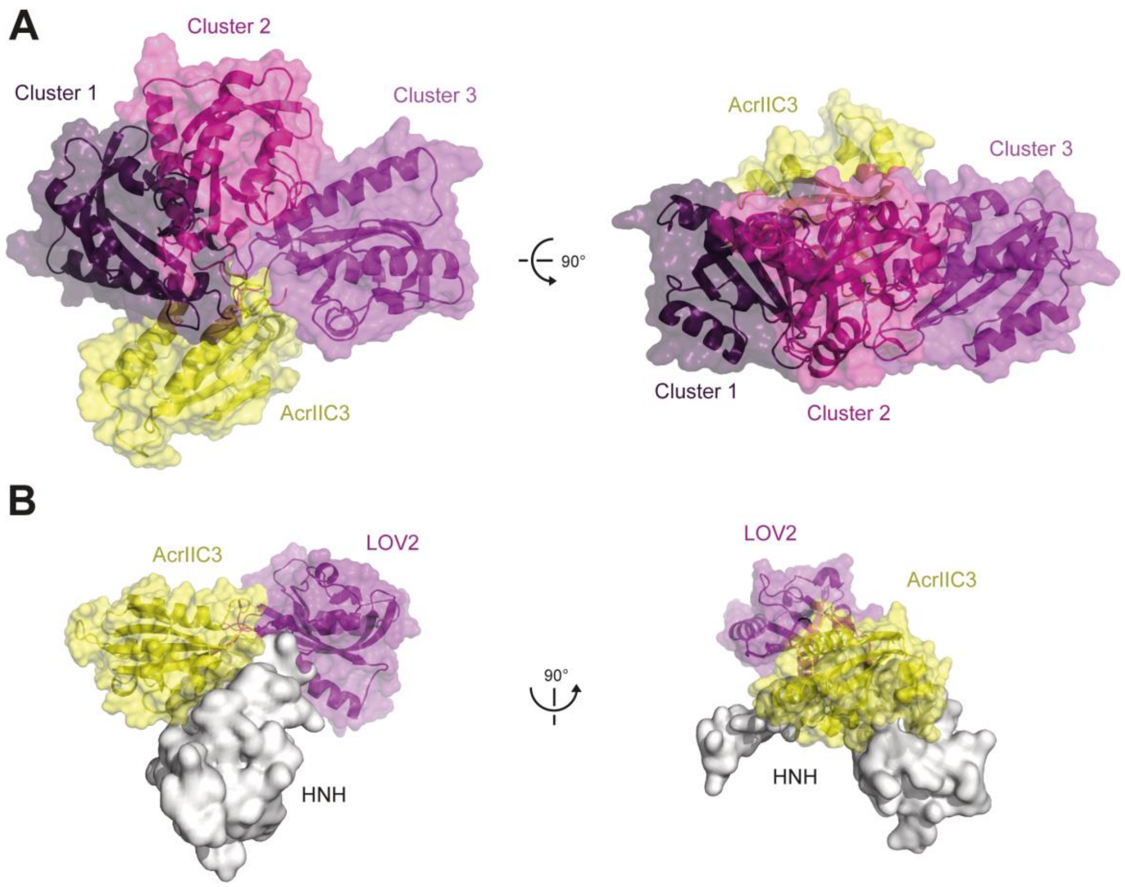

Supplementary Figure S8. Structural models of CN-C3G. (**A**) The three most populated LOV2 conformational clusters as generated by domain assembly simulations are shown. (**B**) In complex with the HNH domain of *Nme*Cas9, only cluster 3 does not show steric clashes. PDB 6J9N, 2V0W.

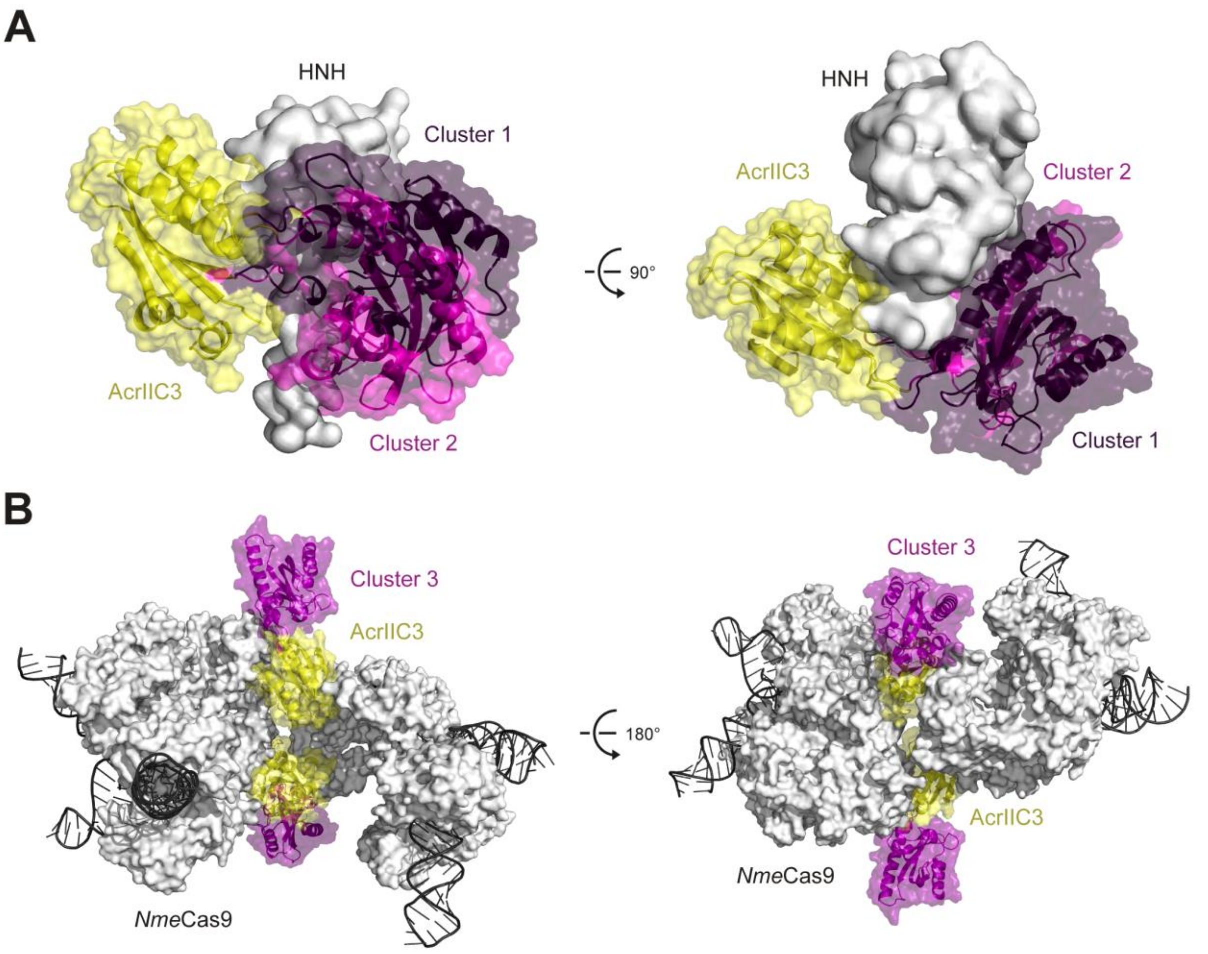

Supplementary Figure S9. (**A**) Two of the three most populated LOV2 conformational clusters sterically clash with the HNH domain. The two most populated cluster of CN-C3 in the HNH-bound form are shown. PDB 6J9N and 2V0W. (**B**) Alignment of the CN-C3 model with LOV2 cluster 3 to the structure of the *Nme*Cas9-AcrIIC3 dimeric complex (3) (PDB 6JE9).

Supplementary Table S1. List of constructs. CMV, cytomegalovirus; AAV, adeno-associated virus; SV40, Simian Virus 40; TK, thymidine kinase; NLS, nuclear localization signal; HA, human influenza hemagglutinin.

#	Name	Description	Source
1	Dual luciferase reporter	SV40 promoter firefly luciferase; TK promoter *Renilla* luciferase; sgRNA targeting firefly gene	(4)
2	h*Nme*Cas9 + sgRNA scaffold (pEJS654 All-in-One AAV-sgRNA-h*Nme*Cas9; Addgene plasmid: #112139)	U1a promoter NLS h*Nme*Cas9 NLS 3xHA; U6 promoter sgRNA scaffold	(5)
3	h*Nme*Cas9 + VEGFA sgRNA (AAV)	U1a promoter NLS h*Nme*Cas9 NLS 3xHA; U6 promoter VEGFA sgRNA	(6)
4	h*Nme*Cas9 + IL2RG sgRNA (AAV)	U1a promoter NLS h*Nme*Cas9 NLS 3xHA; U6 promoter IL2RG sgRNA	(4)
5	h*Nme*Cas9 + FLJ00328 sgRNA (AAV)	U1a promoter NLS h*Nme*Cas9 NLS 3xHA; U6 promoter FLJ00328 sgRNA	(4)
6	h*Nme*Cas9 + AAVS1 sgRNA (AAV)	U1a promoter NLS h*Nme*Cas9 NLS 3xHA; U6 promoter AAVS1 sgRNA	(4)
7	h*Nme*Cas9 + F8 sgRNA (AAV)	U1a promoter NLS h*Nme*Cas9 NLS 3xHA; U6 promoter F8 sgRNA	(4)
8	Wild-type AcrIIC3	CMV promoter AcrIIC3	(6)
9	AcrIIC3 S11-LOV2-F12	CMV promoter AcrIIC3 S11-LOV2-F12	This work
10	AcrIIC3 F12-LOV2-N13	CMV promoter AcrIIC3 F12-LOV2-N13	This work
11	AcrIIC3 N13-LOV2-G14	CMV promoter AcrIIC3 N13-LOV2-G14	This work
12	AcrIIC3 K17-LOV2-V18	CMV promoter AcrIIC3 K17-LOV2-V18	This work
13	AcrIIC3 R33-LOV2-V34	CMV promoter AcrIIC3 R33-LOV2-V34	This work
14	AcrIIC3 V34-LOV2-S35	CMV promoter AcrIIC3 V34-LOV2-S35	This work
15	AcrIIC3 S35-LOV2-I36	CMV promoter AcrIIC3 S35-LOV2-I36	This work
16	AcrIIC3 I36-LOV2-I37	CMV promoter AcrIIC3 I36-LOV2-I37	This work
17	AcrIIC3 A43-LOV2-K44	CMV promoter AcrIIC3 A43-LOV2-K44	This work
18	AcrIIC3 N47-LOV2-A48	CMV promoter AcrIIC3 N47-LOV2-A48	This work
19	AcrIIC3 A48-LOV2-S49	CMV promoter AcrIIC3 A48-LOV2-S49	This work
20	AcrIIC3 S49-LOV2-L50	CMV promoter AcrIIC3 S49-LOV2-L50	This work
21	AcrIIC3 N60-LOV2-D61	CMV promoter AcrIIC3 N60-LOV2-D61	This work
22	AcrIIC3 D61-LOV2-E62	CMV promoter AcrIIC3 D61-LOV2-E62	This work
23	AcrIIC3 S79-LOV2-C80	CMV promoter AcrIIC3 S79-LOV2-C80	This work
24	AcrIIC3 T81-LOV2-G82	CMV promoter AcrIIC3 T81-LOV2-G82	This work
25	AcrIIC3 I83-LOV2-S84	CMV promoter AcrIIC3 I83-LOV2-S84	This work
26	AcrIIC3 E92-LOV-S93	CMV promoter AcrIIC3 E92-LOV-S93	This work
27	AcrIIC3 R97-LOV2-L98	CMV promoter AcrIIC3 R97-LOV2-L98	This work
28	AcrIIC3 L98-LOV2-P99	CMV promoter AcrIIC3 L98-LOV2-P99	This work
29	AcrIIC3 P99-LOV2-V100	CMV promoter AcrIIC3 P99-LOV2-V100	This work
30	AcrIIC3 V100-LOV2-E101	CMV promoter AcrIIC3 V100-LOV2-E101	This work
31	AcrIIC3 L58-LOV2-F59	CMV promoter AcrIIC3 L58-LOV2-F59	This work
32	AcrIIC3 F59-LOV2-N60 (**CN-C3**) (Addgene: #137191)	CMV promoter AcrIIC3 F59-LOV2-N60	This work
33	AcrIIC3 N60-LOV2-D61	CMV promoter AcrIIC3 N60-LOV2-D61	This work

34	AcrIIC3 D61-LOV2-E62	CMV promoter AcrIIC3 D61-LOV2-E62	This work
35	AcrIIC3 E62-LOV2-S63	CMV promoter AcrIIC3 E62-LOV2-S63	This work
36	AcrIIC3 S63-LOV2-P64	CMV promoter AcrIIC3 S63-LOV2-P64	This work
37	AcrIIC3 P64-LOV2-A65	CMV promoter AcrIIC3 P64-LOV2-A65	This work
38	AcrIIC3 F59-LOV2-S63	CMV promoter AcrIIC3 F59-LOV2-S63	This work
39	AcrIIC3 N60-LOV2-S63	CMV promoter AcrIIC3 N60-LOV2-S63	This work
40	AcrIIC3 D61-LOV2-S63	CMV promoter AcrIIC3 D61-LOV2-S63	This work
41	AcrIIC3 F59-LOV2-E62	CMV promoter AcrIIC3 F59-LOV2-E62	This work
42	AcrIIC3 F59-G-LOV2-G-N60 (**CN-C3G**) (Addgene: #137192)	CMV promoter AcrIIC3 F59-G-LOV2-G-N60	This work
43	AcrIIC3 F59-GS-LOV2-SG-N60	CMV promoter AcrIIC3 F59-GS-LOV2-SG-N60	This work
44	AcrIIC3 F59-GSG-LOV2-GSG-N60	CMV promoter AcrIIC3 F59-GSG-LOV2-GSG-N60	This work
45	AcrIIC3 F59-G-LOV2-N60	CMV promoter AcrIIC3 F59-G-LOV2-N60	This work
46	AcrIIC3 F59-LOV2-G-N60	CMV promoter AcrIIC3 F59-LOV2-G-N60	This work
47	AcrIIC3 F59-GS-LOV2-N60	CMV promoter AcrIIC3 F59-GS-LOV2-N60	This work
48	AcrIIC3 F59-LOV2-SG-N60	CMV promoter AcrIIC3 F59-LOV2-SG-N60	This work
49	AcrIIC3 N60-G-LOV2-G-D61	CMV promoter AcrIIC3 N60-G-LOV2-G-D61	This work
50	AcrIIC3 N60-GS-LOV2-SG-D61	CMV promoter AcrIIC3 N60-GS-LOV2-SG-D61	This work
51	AcrIIC3 N60-GSG-LOV2-GSG-D61	CMV promoter AcrIIC3 N60-GSG-LOV2-GSG-D61	This work
52	AcrIIC3 N60-G-LOV2-D61	CMV promoter AcrIIC3 N60-G-LOV2-D61	This work
53	AcrIIC3 N60-LOV2-G-D61	CMV promoter AcrIIC3 N60-LOV2-G-D61	This work
54	AcrIIC3 N60-GS-LOV2-D61	CMV promoter AcrIIC3 N60-GS-LOV2-D61	This work
55	AcrIIC3 N60-LOV2-SG-D61	CMV promoter AcrIIC3 N60-LOV2-SG-D61	This work
56	AcrIIC3 D61-G-LOV2-S63	CMV promoter AcrIIC3 D61-G-LOV2-S63	This work
57	AAV AcrIIC3 (Addgene: # 120301)	AAV vector encoding AcrIIC3	(5)
58	AAV AcrIIC3 F59-LOV2-N60	AAV vector encoding AcrIIC3 F59-LOV2-N60	This work
59	AAV AcrIIC3 F59-G-LOV2-G-N60	AAV vector encoding AcrIIC3 F59-G-LOV2-G-N60	This work
60	HA-AcrIIC3	CMV promoter HA-AcrIIC3	This work
61	HA-CN-C3	CMV promoter HA-CN-C3	This work
62	pBluescript	Empty vector	Invitrogen

Supplementary Table S2. Genomic target sites used for genome editing experiments. Spacer sequences are underlined. PAM motifs are in bold.

Locus	Sequence 5‘ –> 3‘
IL2RG (1)	CTCTTTCTCCTCAAGGAACAATCAGTG**GATT**
FLJ00328 (1)	GGACAGGAGTCGCCAGAGGCCGGTGGTG**GATT**
AAVS1 (1)	ACCCCACAGTGGGGCCACTAGGGACAG**GATT**
F8 (1)	GGTTTCTAGTTGTGACAAGAACACTGGT**GATT**
VEGFA (1)	GCGGGGAGAAGGCCAGGGGTCACTCCAG**GATT**
SLC9A9 (1)	TGGTCTGGGGTACAGCCTTGGCATCAT**GATT**

Supplementary Table S3. List of primers used for genomic PCRs for T7 assay and TIDE sequencing.

Locus	Direction	Sequence 5‘ –> 3‘
IL2RG	Forward	ATGACACTGGTGGGTGTTCAG
	Reverse	TCTTCACCTTGCAGGCTCTCT
FLJ00328	Forward	AGAGGAGCCTTCTGACTGCTGCAGA
	Reverse	AGGTCCTGGCCTTGCCTTCGA
AAVS1	Forward	TGCTTTCTTTGCCTGGACAC
	Reverse	CCTCTCTGGCTCCATCGTAA
F8	Forward	GGGAGAGAACCTCTAACAGAACG
	Reverse	GCTCCAGGTGATGGATCATCAG
VEGFA	Forward	GTGTGCAGACGGCAGTCACTAG
	Reverse	CTCTGCGGACGCTCAGTGAAG
SLC9A9	Forward	GCACTTATTCTGGCCCCTGACTGC
	Reverse	GAGAACCATGGTCTGGGGAAGAAGACC
SLC9A9, off-target	Forward	AGGCCTGGGCTTTATCCA
	Reverse	AGCAGTAGTTCTCAAACTATGT

REFERENCES

1. Amrani, N., Gao, X.D., Liu, P., Edraki, A., Mir, A., Ibraheim, R., Gupta, A., Sasaki, K.E., Wu, T., Donohoue, P.D. *et al.* (2018) NmeCas9 is an intrinsically high-fidelity genome-editing platform. *Genome Biol*, **19**, 214.
2. Bubeck, F., Hoffmann, M.D., Harteveld, Z., Aschenbrenner, S., Bietz, A., Waldhauer, M.C., Borner, K., Fakhiri, J., Schmelas, C., Dietz, L. *et al.* (2018) Engineered anti-CRISPR proteins for optogenetic control of CRISPR-Cas9. *Nat Methods*, **15**, 924-927.
3. Sun, W., Yang, J., Cheng, Z., Amrani, N., Liu, C., Wang, K., Ibraheim, R., Edraki, A., Huang, X., Wang, M. *et al.* (2019) Structures of Neisseria meningitidis Cas9 Complexes in Catalytically Poised and Anti-CRISPR-Inhibited States. *Mol Cell*.
4. Mathony, J., Harteveld, Z., Schmelas, C., Upmeier zu Belzen, J., Aschenbrenner, S., Hoffmann, M.D., Stengl, C., Scheck, A., Rosset, S., Grimm, D. *et al.* (2019) Computational design of anti-CRISPR proteins with improved inhibition potency and expanded specificity. *bioRxiv*, 685032.
5. Ibraheim, R., Song, C.-Q., Mir, A., Amrani, N., Xue, W. and Sontheimer, E.J. (2018) All-in-one adeno-associated virus delivery and genome editing by Neisseria meningitidis Cas9 in vivo. *Genome Biology*, **19**, 137.
6. Hoffmann, M.D., Aschenbrenner, S., Grosse, S., Rapti, K., Domenger, C., Fakhiri, J., Mastel, M., Borner, K., Eils, R., Grimm, D. *et al.* (2019) Cell-specific CRISPR-Cas9 activation by microRNA-dependent expression of anti-CRISPR proteins. *Nucleic Acids Res*, **gkz271**.
